# Thermal Hazard Evaluation of Lauroyl Peroxide Mixed with Nitric Acid 

**DOI:** 10.3390/molecules17078056

**Published:** 2012-07-04

**Authors:** Lung-Chang Tsai, Mei-Li You, Mei-Fang Ding, Chi-Min Shu

**Affiliations:** 1 Department of Safety Engineering, School of Chemical Engineering, Nanjing University of Science and Technology, Nanjing 210094, China; Email: g9410816@yuntech.edu.tw; 2 Department of General Education Center, Chienkuo Technology University, 1, Chieh-Shou N. Rd., Changhua 50094, Taiwan; 3 Department of Applied Chemistry, Providence University, Sha-Lu, Taichung 43301, Taiwan; Email: mfding@pu.edu.tw; 4 Process Safety and Disaster Prevention Laboratory, Department of Safety, Health, and Environmental Engineering, National Yunlin University of Science and Technonlogy, 123, University Rd., Sec. 3, Douliou, Yunlin 64002, Taiwan; Email: shucm@yuntech.edu.tw

**Keywords:** lauroyl peroxide (LPO), nitric acid (HNO_3_), exothermic onset temperature (T_0_), heat of decomposition (ΔH_d_), isothermal time to maximum rate (TMR_iso_)

## Abstract

Many thermal runaway incidents have been caused by organic peroxides due to the peroxy group, –O–O–, which is essentially unstable and active. Lauroyl peroxide (LPO) is also sensitive to thermal sources and is incompatible with many materials, such as acids, bases, metals, and ions. From the thermal decomposition reaction of various concentrations of nitric acid (HNO_3_) (from lower to higher concentrations) with LPO, experimental data were obtained as to its exothermic onset temperature (T_0_), heat of decomposition (ΔH_d_), isothermal time to maximum rate (TMR_iso_), and other safety parameters exclusively for loss prevention of runaway reactions and thermal explosions. As a novel finding, LPO mixed with HNO_3_ can produce the detonation product of 1-nitrododecane. We used differential scanning calorimetry (DSC), thermal activity monitor III (TAM III), and gas chromatography/mass spectrometer (GC/MS) analyses of the reactivity for LPO and itself mixed with HNO_3_ to corroborate the decomposition reactions and reaction mechanisms in these investigations.

## Abbreviations:

E_a_: Activation energy (kJ/mol)
R: Gas constant (J/(mol k))
Q: Heat power (W/g)
T_0_: Exothermic onset temperature (°C)
T: Absolute temperature (K)
∆H_d_: Heat of decomposition (J/g)
TMR_iso_: Isothermal time to maximum rate (min, h, or day)
N: Thermal power or heat production rate (W = J/s)
∆H_iso_: Heat of decomposition under isothermal condition (J/g)

## 1. Introduction

In many organic reactions organic peroxides are used as an initiator. Lauroyl peroxide (LPO) is also employed as a radical source as an initiator or catalyst [1,2,3]. LPO is an oxidizing agent that can ignite organic materials; hence a dangerous fire and explosion risk may inevitably occur if it is not handled properly. Strongly reduced materials, such as sulfides, nitrides, and hydrides, may react explosively. Typical examples include vigorous reactions with other reducing agents, such as with charcoal which sometimes ignites.

Information about organic peroxide mixed with acid or base has recently been reported [4,5,6,7,8]. This study was planned with a view to evaluate the hazard during the period of mixing of LPO contacted with nitric acid (HNO_3_). With calorimetric tests, the hazardous characteristics with respect to specific inorganic acids could be established precisely. The DSC experimental data were obtained of exothermic onset temperature (T_0_), heat of decomposition (ΔH_d_), and other safety parameters for loss prevention of runaway reactions and thermal explosions [7,8,9,10]. The reactivity for LPO and itself mixed with HNO_3_ was evaluated to corroborate the decomposition reactions and reaction mechanisms in these investigations.

Thermal activity monitor III (TAM III) is a micro-isothermal calorimeter that can test thermal behavior under various storage temperatures. We can apply the TAM III to calculate the self-accelerating decomposition temperature (SADT) and isothermal time to maximum rate (TMR_iso_) under related parameters. All sample set-up and data acquisition steps are performed by a devoted software package, TAM III Assistant™, which can also perform most common types of data analyses. In the isothermal mode, the high level of control enables both long and short-term experiments to be performed with excellent baseline stability [11]. The scanning mode operates a linear ramp of temperature with time. Because the instrument records data continuously, also during the temperature change, phase transitions or other temperature-dependent effects will be detected. Since measurement is continuous, there are no breaks in the data collected.

In gas chromatography (GC), a gas or a vaporized sample is introduced and carried along by an inert carrier gas through a long, thin column where the sample components are separated. The components are flushed sequentially from the column and through a detector, and are identified by measuring the time from introduction to detection. The end of the GC column can be coupled directly to the mass spectrometer (MS). The mass spectrometer breaks up constituents into molecular ions and other fragments, which then pass through an electric and/or magnetic field that separates them according to their mass-to-charge ratio. Thus, the GC separates the components within a compound while the MS identifies these components [12,13]. 

GC/MS is a quantitative method [14,15,16,17,18,19] for determination of the components in a complex hydrocarbon mixture. We used GC/MS for the identification and structural analysis of compounds in the reaction of LPO with acids. We compared and analyzed the various compositions of incompatible decomposition by chromatography. We can propose the possible decomposition mechanism and major products under acid-catalyzed situations and acquire information on thermal hazards of LPO with acids of interest.

## 2. Results and Discussion

### 2.1. Thermal Analysis by DSC

**Figure 1:**
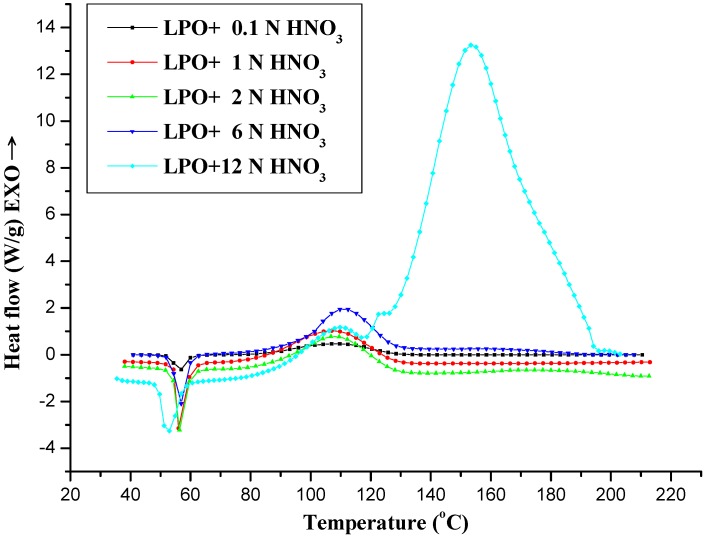
Heat flow *vs.* temperature by the DSC experiments for LPO 95 mass% mixed with 0.1, 1, 2, 6, and 12 N HNO_3_.

HNO_3_ of different concentrations (0.1, 1, 2, 6, and 12 N) was used as contaminant. LPO was mixed with HNO_3_ for DSC tests. The experimental results are displayed in Table 1 and Figure 1. In the investigation, most of the mixing conditions displayed a strong phenomenon: HNO_3_ caused a high degree of thermal hazard during the experiments because the heat of decomposition increased. Especially for HNO_3_ from a concentration of 1 N, in the thermal decomposition process we found the new detonation product 1-nitrododecane. In the 12 N nitric acid contamination even more thermal energy was emitted.

**Table 1:** Calorimetric data from the dynamic scanning experiments of LPO 95 mass% and mixed with HNO_3_ for the total peak of the reaction by DSC.

Sample	Mass (mg)	β (°C/min)	T_max_ (°C)	T_0_ (°C)	ΔH_d_ (J/g)	n	E_a_ (kJ/mol)	ΔH_total_ (mJ/g)
LPO 95 mass% + HNO_3_ (0.1 N, 3.8 mg)	18.3	4	108	68	202	1.17	113	3,682
LPO 95 mass% + HNO_3_ (1 N, 1.6 mg)	10.9	4	108	68	279	1.23	115	4,504
LPO 95 mass% + HNO_3_ (2 N, 1.2 mg)	7.51	4	109	65	600	1.25	129	4,504
LPO 95 mass% + HNO_3_ (6 N, 0.5 mg)	5.37	4	110	60	851	4.32	260	5,024
LPO 95 mass% + HNO_3_ (12 N, 0.8 mg)	6.07	4	160	58	8162	2.36	147	49,550

Early studies of the decomposition peroxides indicated a relatively straightforward mechanism by which the diacyl peroxide broke down by scission of the oxygen-oxygen bond (–O–O–) to render lauroyloxy radicals. When free radicals are produced in solution they are invariably created in pairs. Since small free radicals react with very low activation energies it might be thought that they recombine immediately and no radicals would escape this early “gemination”, period [20]. The homolysis of the major reaction mechanism [21,22] can be explained by the following equations:

#### 2.1.1. Mechanism A


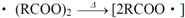
(1)


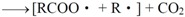
(2)


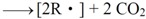
(3)


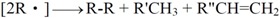
(4)

Accordingly, the initial step (1–4) in the reaction is to generate the lauroyloxy radical; decarboxylation [23] of the lauroyloxy radical is a very rapid process. The decarboxylation of the lauroyloxy radical generates an undecyl radical singlet pair. From this mechanism and GC/MS analysis, we obtained hendecane, hendecene, and a cage product that undergoes a recombination into docosane.

#### 2.1.2. Mechanism B


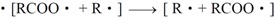
(5)


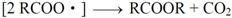
(6)


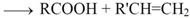
(7)

In the second step (5–7), there is an intersystem crossing to the radical pair, then the radicals are combined together to yield undecyl ester. The presence of acid furnishes another mechanism pathway. Here, [RCOO‧], free radicals integrate with hydrogen producing dodecanoic acid; the secondary reaction that occurred formed the olefin hendecene.

#### 2.1.3. Mechanism C


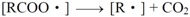
(8)


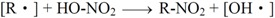
(9)


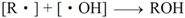
(10)

According to the DSC alignment, when LPO was added to 1 N HNO_3_ in the thermal decomposition process from GC/MS detector analysis, we found 1-nitrododecane. It means that its mechanism (8–10) was complex and showed two consecutive exothermic peaks in the overall reactions. Nitro compounds are organic compounds that contains one or more nitro functional groups (–NO_2_). They are often highly explosive, especially when the compound contains more than one such nitro group. The presence of impurities or improper handling can trigger a violent exothermic decomposition. 

The onset temperature of LPO is 50 °C [7]; when LPO is decomposed by thermal decomposition under the high concentration of 12 N HNO_3_, the [R‧] will be generated. At the same time, [R‧] will react with 12 N HNO_3_, and produce a great deal of nitro compound, that emits a good deal of thermal energy. The results are illustrated in Figure 1. The flash point of 1-nitrododecane was 107 °C [24], when unstable and active peroxy group –O–O– was cleaved and at the same time the flash point of 1-nitrododecane was reached, that emitted enormous amounts of energy. Many thermal runaway incidents have been caused by organic peroxides and the degree of thermal hazard increases significantly if there are acid contaminants. 

### 2.2. Thermal Decomposition Analysis of LPO Mixed with Inorganic Acids by TAM III

A few calorimetric methods can be utilized to test runaway reactions and calculate the kinetic parameters, such as frequency factor (A), apparent activation energy (E_a_), reaction order (n). Designing a suitable vessel and realizing the basic characteristics of a runaway reaction, such as kinetic and safety parameters (TMR_iso_, SADT) for LPO [7], are a prevailing way of reducing the consequences associated with the above risks in an emergency. TAM III was employed to determine isothermal reaction behaviors. Isothermal modeling is extensively used in evaluating the runaway reactions, requiring detailed consideration of several competing and often interacting heat transfer, mass-transfer, and thermodynamic processes. In this study, we elucidated a variety of basic thermal hazard characteristics for LPO with HNO_3_ by using the TAM III tests. 

**Figure 2:**
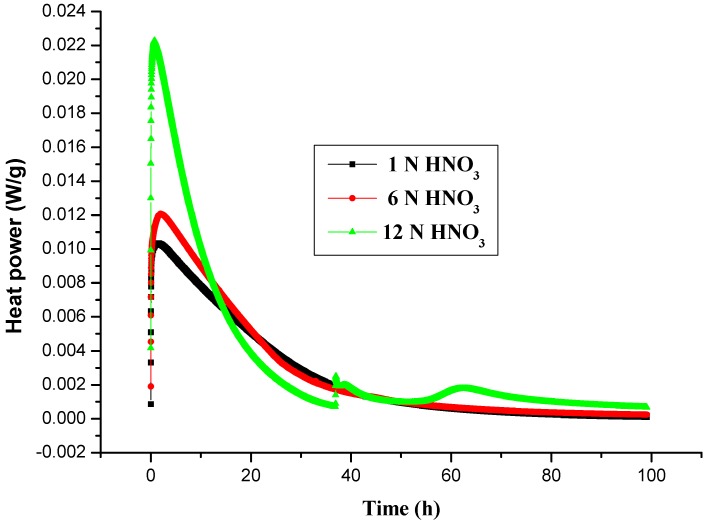
Heat flow *vs.* time for the thermal decomposition of 95 mass% LPO with 1, 6, and 12 N HNO_3_ at 60 °C by TAM III tests.

Table 2, Table 3, Table 4 and Table 5 present the thermal runaway decomposition of LPO mixed with the HNO_3_ under four isothermal temperatures. Figure 2 presents the heat flow *vs.* time for the thermal decomposition of 95 mass% LPO with 1, 6, and 12 N HNO_3_, at 60 °C by TAM III tests. From the investigations, as the isothermal temperature increased, the time for emergency response became significantly shorter. As for the effects of 12 N HNO_3_, the heat flow at 50 °C was about 0.0048 W/g and heat of isothermal (∆H_iso_) was about 2,060 J/g for the effects of 6 N HNO_3_, the heat flow was about 0.0022 W/g and ∆H_iso_ was about 1,013 J/g, for the effects of 1 N HNO_3_, the heat flow was about 0.0027 W/g, and ∆H_iso_ was about 793 J/g, when the concentration of HNO_3_ (12 N) was decreased to HNO_3_ (1 N), the degree of hazard was lessened significantly. Figure 2 presents the heat flow *vs.* time for the thermal decomposition of 95 mass% LPO with 1, 6, and 12 N HNO_3_, at 60 °C by TAM III tests. TMR_iso_ was 11.6, 9.51, and 4.01 h under isothermal conditions of 50 °C for 1, 6, and 12 N HNO_3_. LPO mixed with HNO_3_ [4,6] can produce the detonation product of 1-nitrododecane, which is a novel finding. In TAM III tests, we investigated LPO mixed with 12 N HNO_3_ and found it can emit a large amount of heat at low temperature, but under higher isothermal condition the thermal reaction started before the samples reached a condition of the thermal equilibrium. In our study of LPO from VSP2 [7], LPO did melt at about 50 °C, and the autocatalysis behavior appeared, correspondingly. When LPO is dissolved completely, it would result in an instantaneous runaway, and generate huge pressure and huge amounts of heat.

**Table 2:** Experimental data by TAM III tests for 95 mass% LPO with various concentrations of HNO_3_ at 50 °C.

Sample	Mass/(LPO/contaminant) (mg)	Cell	TMR_iso_ (min)	Heat power ∆H_iso_ (W/g) (J/g)
LPO + 1 N HNO_3_	52.8/15.2	Glass	696	0.0027 793.07
LPO + 6 N HNO_3_	52.7/24.6	Glass	570.6	0.0022 1,013.04
LPO +12 N HNO_3_	52.3/15.2	Glass	240.6	0.0048 2,059.98

**Table 3:** Experimental data by TAM III tests for 95 mass% LPO with various concentrations of HNO_3_ at 60 °C.

Sample	Mass/(LPO/contaminant) (mg)	Cell	TMR_iso_ (min)	Heat power ∆H_iso_ (W/g) (J/g)
LPO + 1 N HNO_3_	59.8/16.0	Glass	94.70	0.0103 894.12
LPO + 6 N HNO_3_	57.4/13.3	Glass	62.04	0.012 976.21
LPO +12 N HNO_3_	52.3/15.2	Glass	38.35	0.023 1,203.64

**Table 4:** Experimental data by TAM III tests for 95 mass% LPO with various concentrations of HNO_3_ at 70 °C.

Sample	Mass/(LPO/contaminant) (mg)	Cell	TMR_iso_ (min)	Heat power ∆H_iso_ (W/g) (J/g)
LPO + 1 N HNO_3_	57.4/14.2	Glass	12.09	0.046 872.0
LPO + 6 N HNO_3_	56.4/10.5	Glass	17.14	0.043 952.8
LPO +12 N HNO_3_	57.1/13.7	Glass	12.8	0.063 1,072.05

**Table 5:** Experimental data by TAM III tests for 95 mass% LPO with various concentrations of HNO_3_ at 80 °C.

Sample	Mass/(LPO/contaminant) (mg)	Cell	TMR_iso_ (min)	Heat power ∆H_iso_ (W/g) (J/g)
LPO + 1 N HNO_3_	60.1/11.9	Glass	8.58	0.13 573.06
LPO + 6 N HNO_3_	60.6/10.06	Glass	10.62	0.12 734.08
LPO + 12 N HNO_3_	60.1/8.5	Glass	8.07	0.15 655.04

In the meantime, LPO was attacked by high concentration of HNO_3_, and then the unstable structure was cleaved and released much more energy. Compared with the heat flow *versus* time for the thermal decomposition of 1, 6, and 12 N HNO_3_ mixed with LPO, the heat flow was quite obviously different between acids. TMR_iso_ under isothermal conditions of 50, 60, 70, and 80 °C for acids are demonstrated in Table 2, Table 3, Table 4 and Table 5. Under isothermal conditions of 70 and 80 °C for LPO mixed acids, there is only 10 to 15 min to respond to the accident.

#### 2.2.1. The Calculation of Thermokinetic Parameters

**Figure 3:**
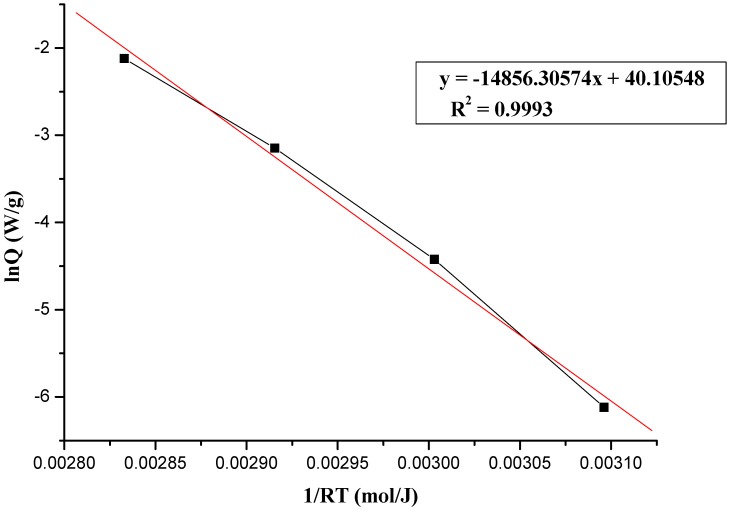
Determination of activation energy from the slope of lnQ *vs.* 1/(RT) for the thermal decomposition of 95 mass% LPO with 1 N HNO_3_ at 50, 60, 70, and 80 °C by TAM III tests.

**Figure 4:**
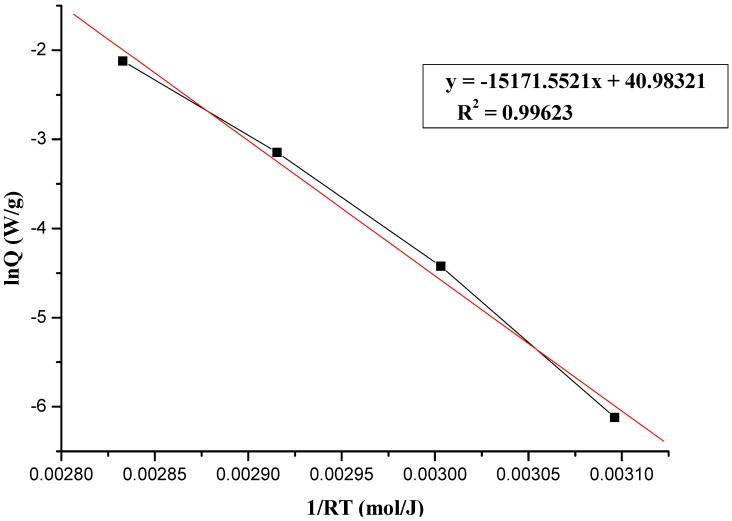
Determination of activation energy from the slope of lnQ *vs.* 1/(RT) for the thermal decomposition of 95 mass% LPO with 6 N HNO_3_ at 50, 60, 70, and 80 °C by TAM III tests.

**Figure 5:**
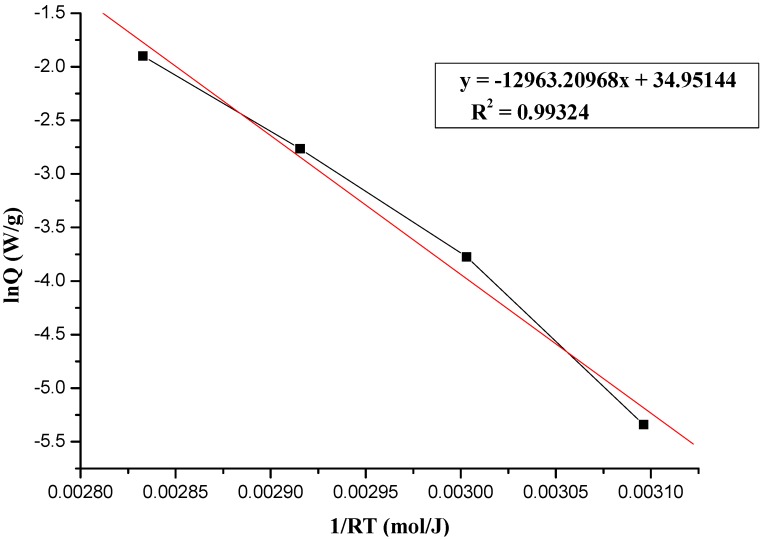
Determination of activation energy from the slope of lnQ *vs.* 1/(RT) for the thermal decomposition of 95 mass% LPO with 12 N HNO_3_ at 50, 60, 70, and 80 °C by TAM III tests.

The results of E_a_ for LPO and with inorganic acid by employing the Arrhenius equation are as shown in Equation (11) [25,26,27]:


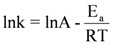
(11)

where A, E_a_, R, and T represent frequency factor, activation energy, gas constant (R = 8.314 J/(mol K), and absolute temperature, respectively. The plot of lnk *vs.* 1/T is expected to be a straight line; the Arrhenius kinetic parameters, E_a_ can be calculated from the plot accordingly to Figure 3, Figure 4 and Figure 5, which show the determination of activation energy from the slope of lnQ *vs.* 1/(RT) for the thermal decomposition of 95 mass% LPO with 1, 6, and 12 N HNO_3_, at 50, 60, 70, and 80 °C by TAM III tests. As calculated from the Arrhenius equation, the value of E_a_ for the thermal decomposition of 95 mass% LPO with 1, 6, and 12 N HNO_3_ at 50, 60, 70, and 80 °C was 124.47, 126.14, and 107.78 J/mol, respectively.

## 3. Experimental

### 3.1. Sample Preparations

95 mass% LPO was directly purchased from the Fluka Co., and both density and concentration were measured. Then, LPO was stored in a refrigerator at 4 °C. Deionized water (H_2_O) was used as the diluent in preparing different concentrations of commonly used inorganic acids, which were selected to combine with LPO in this study for studying its incompatible reactions. The typical reactions of LPO with 0.1, 1, 2, 6, and 12 N HNO_3_ were selected for comparative study.

### 3.2. Differential Scanning Calorimetry (DSC)

Dynamic scanning experiments were performed on a Mettler TA8000 system coupled with a DSC 821^e^ measuring test crucible (Mettler ME–26732) that could withstand relatively high pressure of about 100 bars. STAR^e^ software was employed for acquiring thermal curves. To have a better thermal equilibrium, the heating rate (β) was chosen at 4 °C/min. The test cell was sealed manually by a special tool equipped with Mettler’s DSC. We conducted dynamic scanning by starting the programmed setting. The range of temperature rise was chosen from 30 to 300 °C for each condition of experiments. This apparatus could adequately measure the heat flow, and then via experimental data to calculate the T_0_, TMR_iso_, and ΔH_d_ by software imbedded in the interior function of DSC.

### 3.3. Gas Chromatography/Mass Spectrometer (GC/MS)

Thermal decomposition of LPO with acids and its decomposition products was determined by GC/MS [14]. It was an Agilent Technologies 6890N model equipped with a split/splitless injector. The details of the column are: DB-WAX; Length 30; I. D. 0.25 mm; Film (μm) 0.25. The injector temperature was 280 °C and the detector temperature was 250 °C. The GC/MS oven temperature was programmed from 60 °C kept at 1 min then 20 °C/min to 250 °C, which was held for 15 min. Here, helium was used as carrier gas at a flow rate of 1.2 mL/min.

### 3.4. Thermal Activity Monitor III (TAM III)

Isothermal microcalorimetry (TAM III) is generally applied on a range of products for thermal measurements originally manufactured by Thermometric in Sweden. TAM III was used to investigate the runaway reaction of LPO mixed with HNO_3_ at 50, 60, 70, and 80 °C. The absolute temperature could be adjusted to within 0.02 K; while operating in isothermal mode, the bath mean temperature fluctuations were within 10^–5^ K. The maximum scanning rate is ±2 K/h for nearly chemical and physical equilibrium. We used the software of TAM III Assistant™ to govern the thermostat. The thermostat liquid is mineral oil with a total volume of 22 L, and the temperature range of the thermostat is 15–150 °C when mineral oil is employed. This study investigated LPO mixed with HNO_3_ solution under a specified isothermal condition. Under the isothermal conditions, a series of LPO homolytic scissions were carried out, and the reaction and thermal decomposition of LPO individually mixed with HNO_3_ solution were confirmed completely.

## 4. Conclusions

Many organic peroxides have been defined as hazardous materials, and LPO is among them. From our study of the thermal decomposition of LPO and acids, these have thermal hazard potentials due to their unstable and sensitive characteristics. According to the experimental results, LPO is very sensitive to inorganic acids (here HNO_3_), especially at high concentrations (12 N). 

We believe that many peroxides in storage and in transport may potentially be contaminated by acids or other pollutants. Instantaneous decomposition or combustion of such unstable substances may engender explosions and create thermal hazards. Through the results of this study, we could provide information on the hazards and instruct the relevant staff on how to forestall and mitigate an accident before or during the period of runaway triggered by LPO along with the above-mentioned contaminants. 
